# FISH and Chimps: Insights into Frequency and Distribution of Sperm Aneuploidy in Chimpanzees (*Pan troglodytes*)

**DOI:** 10.3390/ijms221910383

**Published:** 2021-09-27

**Authors:** Charlotte Guyot, Marlène Gandula, Wendy Noordermeer, Céline François-Brazier, Rosemary Moigno, Julien Bessonnat, Sophie Brouillet, Magali Dhellemmes, Marie Bidart, Christophe Arnoult, Véronique Satre, Charles Coutton, Guillaume Martinez

**Affiliations:** 1 Hôpital Couple-Enfant, Centre Hospitalier Universitaire de Grenoble, UM de Génétique Chromosomique, 38000 Grenoble, France; cguyot2@chu-grenoble.fr (C.G.); mgandula@chu-grenoble.fr (M.G.); vsatre@chu-grenoble.fr (V.S.); ccoutton@chu-grenoble.fr (C.C.); 2 Le PAL, Saint-Pourçain-sur-Besbre, 03290 Dompierre-sur-Besbre, France; wendynoordermeer@lepal.com (W.N.); cfrancois-brazier@hotmail.fr (C.F.-B.); rosemaryveto@lepal.com (R.M.); 3 Hôpital Couple-Enfant, Centre Hospitalier Universitaire de Grenoble, Centre Clinique et Biologique d’Assistance Médicale à la Procréation, Centre D’étude Et De Conservation Des Œufs Et Du Sperme Humains (CECOS), 38000 Grenoble, France; jbessonnat@chu-grenoble.fr; 4 Université de Montpellier, EmbryoPluripotency, DEFE, INSERM 1203, Hôpital Arnaud de Villeneuve, CHRU Saint-Eloi, 80 Avenue Augustin Fliche, CEDEX 05, 34295 Montpellier, France; s-brouillet@chu-montpellier.fr; 5 Genetic Epigenetic and Therapies of Infertility, Institute for Advanced Biosciences INSERM U1209, CNRS UMR5309, 38000 Grenoble, France; magali.dhellemmes@univ-grenoble-alpes.fr (M.D.); mbidart@chu-grenoble.fr (M.B.); christophe.arnoult@univ-grenoble-alpes.fr (C.A.); 6 Station de Primatologie, UPS 846, CNRS, 13790 Rousset, France

**Keywords:** chromosome, cytogenetic, infertility, non-human primate, reproduction, spermatozoa

## Abstract

Numerical chromosomal aberrations in sperm are considered to be a major factor in infertility, early pregnancy loss and syndromes with developmental and cognitive disabilities in mammals, including primates. Despite numerous studies in human and farm animals, the incidence and importance of sperm aneuploidies in non-human primate remains mostly undetermined. Here we investigated the incidence and distribution of sperm aneuploidy in chimpanzees (*Pan troglodytes*), the species closest to human. We identify evolutionary conserved DNA sequences in human and chimpanzee and selected homologous sub-telomeric regions for all chromosomes to build custom probes and perform sperm-FISH analysis on more than 10,000 sperm nuclei per chromosome. Chimpanzee mean autosomal disomy rate was 0.057 ± 0.02%, gonosomes disomy rate was 0.198% and the total disomy rate was 1.497%. The proportion of X or Y gametes was respectively 49.94% and 50.06% for a ratio of 1.002 and diploidy rate was 0.053%. Our data provide for the first time an overview of aneuploidy in non-human primate sperm and shed new insights into the issues of aneuploidy origins and mechanisms.

## 1. Introduction

The number of species at risk of extinction is currently nearly 1000 times the normal rate of extinction through evolution [1,2]. Primates are not spared and ~75% of species have declining populations worldwide [3]. To support species conservation and prevent inbreeding depression, new approaches have emerged incorporating genetics and/or reproductive biology technologies [4]. For instance, a genetic approach using non-invasively collected samples of a population allows an accurate characterization of its size, dynamics, dispersion distance and gene flow, but also the determination of group membership, movements, and relatedness at individual scale [5,6]. Active genetic management of fragmented populations is then achievable. Genetic diversity can be introduced by individual relocation or via medically assisted reproduction techniques (ART), predominantly vaginal or intrauterine inseminations [1]. Beyond genetic rescue situations, ART also allow for the conservation of genetic material and detection of potential inbreeding (reviewed in [7,8]). One limitation to the development of new approaches is the lack of data for several aspects of primate reproduction and further research combining procreation and genetics are needed to advance current understanding of the primate reproductive system [9].

Numerical chromosomal aberrations in sperm are considered to be a major factor in infertility, early pregnancy loss and syndromes with developmental and cognitive disabilities in mammals [10,11,12,13,14], including primates [15,16,17,18,19,20]. Sperm aneuploidy analysis have now been pursued for decades using hybridization in situ fluorescence (FISH) techniques, namely spermFISH. Although most of these studies were focus on humans, many mammalian species were investigated like cattle [21,22,23] and river buffalo [24], horse [25,26], pig [27,28], goat [24], sheep [24] and dog [29]. Nevertheless, literature on sperm aneuploidy in non-human primates remains scarce and sperm aneuploidy rates were reported only in two publications. O’Brien and collaborators [30] first published successful hybridization of probes directed against human chromosomes in *Pan troglodytes*, *Papio hamadryas* and *Callithrix jacchus* spermatozoa. Then, Froenicke and collaborators [31] truly paved the way of sperm aneuploidy analysis in non-human primates by reporting a six-chromosome sperm-FISH assay with probes directed against regions with high content of evolutionarily conserved DNA sequences in *Macaca mulatta*. However, so far, the incidence and importance of sperm aneuploidies in non-human primate remains mostly undetermined.

Two factors are mainly responsible for this lack of investigation. First, excluding species used for biomedical research such as *Macaca mulatta*, it is logistically challenging to collect primate semen, and when possible, it is foremost used for species conservation purposes [32]. Second, spermFISH analysis is extremely time consuming. The low incidence of sperm aneuploidy requires a minimum of 10,000 nuclei per chromosome to be analyzed for statistical significance. Although automated or semi-automated approaches have been implemented over the years [33,34,35,36,37] making the analysis more accessible, it still represents a huge amount of technical time. Even in human clinical practice, only a few chromosomes are usually studied, commonly chromosomes 13, 18, 21, X and Y, which are implicated in aneuploidies compatible with survival. To overcome these limitations, we used thawed straws from a control series of chimpanzee (*Pan troglodytes*) non-invasive sperm preservations, and developed a hybridization technique that prevents sperm heads swelling and improves signal quality. The analysis process is thus accelerated and allows to have a first glimpse of the chimpanzee sperm aneuploidy rates.

Given their proximity to humans and the limited data available on them, chimpanzees would be an appropriate starting point for the evaluation of sperm aneuploidy in non-human primates. We report here for the first time the frequency and distribution of sperm aneuploidy for all chromosomes of two 48,XY adult chimpanzees. We also specify their sperm parameters, their karyotypes with regard to a human one, provide all the clones used for hybridization, validate an improved FISH protocol for non-human primate spermatozoa, compare their aneuploidy rates with those of the other primates and discuss the implications of these results for the understanding of sperm aneuploidies.

## 2. Results

### 2.1. Chimpanzee Biological Data

**Figure 1:**
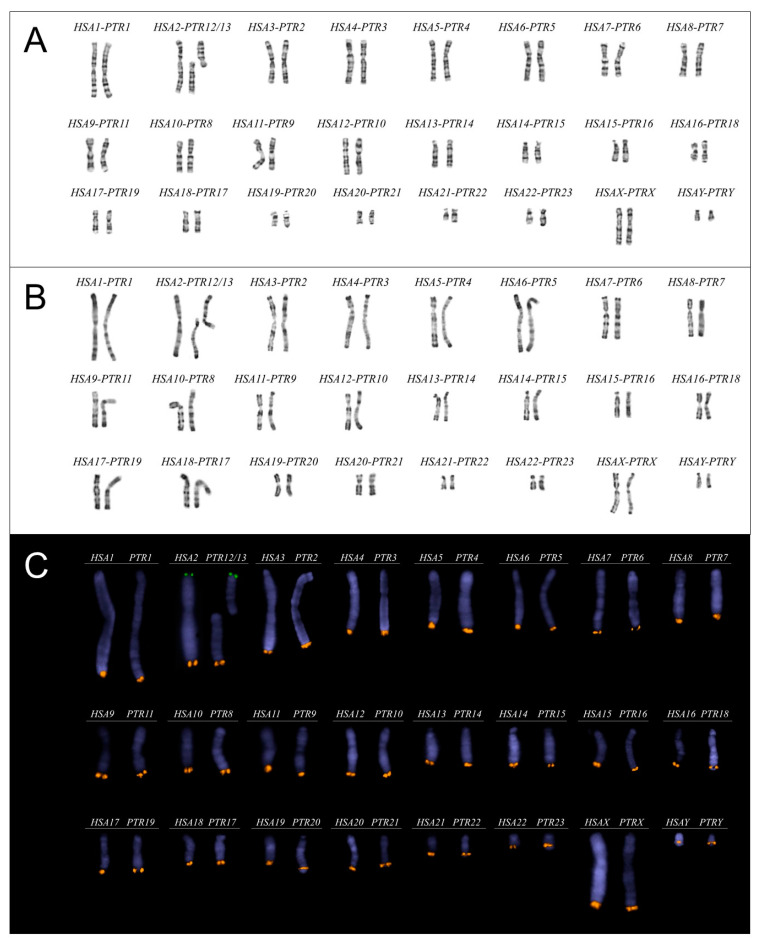
(**A**) G-banding and (**B**) R-banding karyogram of Homo sapiens and Pan troglodytes. (**C**) Fluorescent karyogram with positions of all probes used for chromosome enumeration. For each pair, human chromosomes (HSA) are positioned on the left and chimpanzee chromosomes (PTR) on the right. Probes from the short arm of human chromosome 2 and chimpanzee chromosome 13 were labeled in Spectrum Green and the others in Spectrum Orange (the color combinations used for the analyses are listed in the Material and Methods section).

We first confirmed that the two males had no spermogram or karyotype anomaly, these results being consistent with their status as active breeders. Sperm parameters are provided in Table 1 with both males displaying values in the literature normal range for this species, including normal sperm morphology with 84% and 79% of normal form. Both chimpanzees displayed a normal 48,XY karyotype free of any visible structural or numerical chromosomal rearrangement within the limit of the karyotype resolution in G-banding (Figure 1A) and R-banding (Figure 1B).

### 2.2. DNA Probes

We used UCSC Genome Browser [38] to identify evolutionary conserved DNA sequences in human and chimpanzee and selected homologous sub-telomeric regions in long arms of all chromosomes with the exception of chromosome 2. As human chromosome 2 originated from the telomeric fusion of two ancestral primate chromosomes corresponding to chimpanzee chromosomes 12 and 13 [39], we selected two sub-telomeric conserved regions on the short and long arms of the human chromosome 2 to be able to analyze all chimpanzee chromosomes. For each selected region, two to nine (mean = 6) bacterial artificial chromosome (BAC) DNAs, spanning genomic distances from 520 to 1918 kbp (mean = 1197 kbp), were pooled to be used as FISH probes. All BACs are listed in Supplemental Table S1 and were successfully validated on both human and chimpanzee mitosis with a hybridization rate of 100%. Each probe set displays a strong independent signal on its chromosome with no visible cross-hybridization on any other chromosome (Figure 1C). Each probe can then be labeled with Spectrum Green (SG), Spectrum Orange (SO) or Spectrum Aqua (SA) fluorochromes to obtain different color combinations required to perform the analysis.

### 2.3. Sperm Aneuploidy

**Figure 2:**
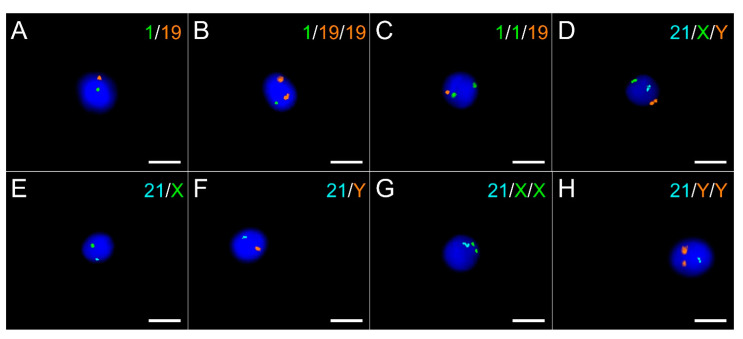
FISH analysis of chimpanzee spermatozoa with two (**A**–**C**) and three-color (**D**–**H**) in situ hybridization. Panels (**A**) to (**C**) are labeled with 1 Spectrum Green (SG)/19 Spectrum Orange (SO) probes with (**A**) displaying a normal nuclei, (**B**) a disomy of chromosome 19 and (**C**) a disomy of chromosome 1. Panels (**D**–**H**) are labeled with 21 Spectrum Aqua (SA)/X SG/Y SO probes with (**E**,**F**) displaying normal 21, X and 21, Y nuclei, and (**D**,**G**,**H**) displaying disomy of sex chromosomes. Scale = 10 µM.

As for FISH experiments on mitosis, all probe sets give strong independent signals for all chromosomes in spermatozoa as illustrated in Figure 2. A total of 120,972 sperm nuclei were analyzed to assess aneuploidy rates in chimpanzees, with an average of 10,081 sperm nuclei per probe set (range 10,003–10,298). Disomy rates per chromosome are displayed in Table 2. The mean autosomal disomy rate is 0.057 ± 0.02% with a range of 0.02 (for chromosomes 8 and 17, corresponding to human chromosomes 10 and 18) to 0.09 (chromosome 16, corresponding to human chromosome 15). The gonosomes disomy rate is 0.198% and the total disomy rate is 1.497%. The proportion of X or Y gametes is respectively 49.94% and 50.06% for a ratio of 1.002. Diploidy rate is 0.053% with a range from 0.03% (probes set 18/19) to 0.098% (probes set 8/14) according to the chromosome set (Table 3).

### 2.4. Interspecies Comparison of Sperm Aneuploidy Rates

In order to compare the chimpanzee and human sperm aneuploidy rates, we conducted a literature review on human sperm aneuploidy. To this end, the PubMed/MEDLINE and Google Scholar databases were screened from inception to June 2021 using combinations of terms (including terms “Aneuploidy”, “Human”, “Spermatozoa”, “spermFISH”, and “Chromosome abnormalities”). Additional studies were extracted from the references of the full-text articles. Data contained in the reviews were cited via their original articles. Full texts of potentially relevant articles were retrieved and evaluated for inclusion. Only multicolor-FISH studies with a minimum of 5 donors, strict scoring criteria, and a minimum of 10,000 nuclei counted per probe set were included. After searching the databases, 1441 abstracts were reviewed, and 143 full-text articles were assessed for eligibility. The articles that did not contain relevant data for our topic or did not meet our inclusion criteria were excluded and data from 34 articles were included. The following details were extracted from each source: study authors, publication year and disomy rates per chromosome. Literature review results are reported in Supplementary Table S2 and human disomy rates are displayed in Table 4 alongside chimpanzee and macaque rates to allow interspecies comparisons.

The observed rates of spontaneous chromosomal aneuploidies in chimpanzees (mean autosome: 0.057%; mean gonosome: 0.198%; total = 1.497%) are similar to those in macaques (mean autosome: 0.058%; mean gonosome: 0.190%; predicted total = 1.292%) for the few chromosomes studied and coincide with the lowest reported incidences in humans (autosome range: 0.04–0.37%, mean: 0.15; predicted total = 3.69%). The incidence of autosomal aneuploidies in chimpanzees and macaques are about 3 times lower than in humans. Similar to humans and macaques, chimpanzee sex chromosomes are more frequently involved in aneuploidies than autosomes. Gonosomal anomalies are 3.5-fold higher in Pan troglodytes compared to autosomal aneuploidies, 1.7-fold higher in Homo sapiens, and 3.3-fold in Macaca mulatta. However, the gonosomal aneuploidies are about 1.4-fold lower in primates than in human. Concerning diploidy, the chimpanzee rate is 0.053%, similar to the 0.050% of Macaca Mulatta, both six-times inferior to the 0.310% of human.

## 3. Discussion

This study provides a first insight into the incidence of chromosomal aneuploidies in chimpanzee sperm. We showed that, like all the other studied mammals, chimpanzees produce a proportion of aneuploid spermatozoa even when they are healthy and fertile. The presence of disomic spermatozoa for all chromosomes proves that all chimpanzee chromosomes are subject to non-disjunction during spermatogenesis.

We reported little variation between all the autosomal disomies incidence for chimpanzee which is also reported in *Macaca mulatta* [31]. In contrast, some human autosomes are more prone to non-disjunction than others [40]. It has been suggested that the presence of nucleolus organizing regions (NORs) on the short arms of acrocentric chromosomes would predispose them to non-disjunction [41,42] but we did not find a significant difference between the incidence rates of acrocentric chromosomes versus the others in chimpanzee (student *t*-test, *t* = −0.66, df = 10.97, *p*-value = 0.52, NS). The chromosome size is a factor in mitotic non-disjunction, it has also been proposed to have an impact during meiosis [43]. Again, we did not observe a significant difference when comparing the largest chimpanzee chromosomes to the smallest (student *t*-test, *t* = –0.25, df = 21, *p*-value = 0.80, NS). Very few data are available on the products of miscarriages in primates, so it is difficult to ascertain the origin of aneuploidies, but as in humans, there is a possibility of a greater preponderance of female non-disjunctions.

Interestingly however, all three species displayed a similar two- to three-fold higher incidence of gonosome aneuploidy. Several hypotheses have already been proposed to explain the prevalence of gonosome aneuploidies in human [44] but it has not yet been determined whether it is a lack of pairing or of recombination that leads to the non-disjunction of the bivalent. Here we hypothesize the existence of mechanisms that would be common to all primates to explain the potential non-disjunction of all chromosomes and the universal instability of the sex chromosomes bivalent, and of an additional potential human-specific mechanism that would explain his higher disomy rate. It is also possible that the initial non-disjunction rate is the same for all chromosomes but that these are subject to a different regulation, presenting a more or less important tolerance according to the chromosomes. A more permissive human spermatogenesis or stronger regulatory mechanisms in primates that would prevent the evolution of disomic spermatozoa could explain why chimpanzees have a total disomy incidence close to that of macaques but far inferior to that of humans.

Concerning the diploidy rate, it is similar in chimpanzee (0.053%) and macaque (0.050%) and about six-times lower than in human. We could also hypothesize that some regulatory controls are more effective in these two species than in human but there is no evidence nowadays.

Our understanding of the genetic mechanisms contributing to the increase in sperm aneuploidy remains limited, but several human studies have highlighted the likely existence of meiotic controls that would block the proliferation of a portion of aneuploid cells [45,46,47,48]. Investigating aneuploidy in chimpanzee meiosis II spermatocytes and comparing them to the levels observed in spermatozoa would allow us to determine if such a meiotic control exists in chimpanzees. Our hypothesis is that all great apes could potentially have a similar incidence of aneuploidy during meiosis but different effective meiotic checkpoints.

In human, some morphological abnormalities of spermatozoa have been associated with higher rates of aneuploidy in spermatozoa [49] and in preimplantation embryos [50]. Alongside humans, gorillas are the only other primates to exhibit extreme sperm pleiomorphism. As it has not yet been determined whether this phenomenon is functionally relevant, plays a key role in fertility, or is simply the result of a lack of pressure from sperm competition in those two species [9], it would be of great value to explore sperm aneuploidy in gorillas. Whether they display high rates of sperm aneuploidy like humans or low rates like chimpanzees could influence the debate about the origin and relevance of sperm pleiomorphism in these species.

Although the origins of gamete aneuploidy are still debated, a portion of aneuploidy is thought to be due to defects in reciprocal exchange (or crossing over) between homologous chromosomes, resulting in poor segregation (or non-disjunction) of chromosomes during the first or second meiotic division [51,52]. Crossing-over are non-randomly distributed along the genome in hot spot intervals of 1 to 2 kb which are estimated to be 25,000 in the whole human genome against only 8037 in the chimpanzee although the two species exhibit similar recombination rates [53] and comparable genome size. This divergence, which would be in part the result of a modification in the zinc fingers of Prdm9, a protein involved in the control of hot spot activity [54], could be at the origin of the differences in aneuploidy rates between the two species. Very interestingly, the number of hot spots in gorillas is estimated to be 22,012 [53], which is much closer to humans than chimpanzees although they are not the closest relative. These human-gorilla similarities may originate from a portion of the human genome, estimated to be about 15%, that is much closer to the Gorilla genome than to the Chimpanzee one [55]. This emphasizes again the need for larger studies on great ape reproduction to help the comprehension of mechanisms still poorly understood in humans.

The data reported in this study came from the pooled sperm of two adult chimpanzees and allow having a first insight into chimpanzee sperm numerical abnormalities. Our results are however not sufficient to draw conclusions for the whole species. In humans, many factors like genetic [56], age [57], food [58], disease [59], inbreeding degree [60], and environmental components such as exposure to heat [61], pollutants or chemical products [62,63,64,65,66] can influence the incidence of aneuploidies resulting in large inter-individual differences [67,68,69]. Non-human primates could also potentially be pressured by some of these factors. Future studies are needed and will be eagerly awaited to report more individuals and to best determine the incidence of sperm aneuploidy in chimpanzees, taking into account the elements influencing its occurrence. Situations in which it is possible to recover sperm from great apes without human intervention are scarce and every opportunity should be taken to learn more from them.

## 4. Materials and Methods

### 4.1. Animals and Sample Collection

All samples were obtained from two adult male chimpanzees of known fertility housed in Le Pal, French zoo member of the European Association of Zoos and Aquaria (EAZA). The best veterinary care practices are ensured for the animals, in accordance with the protocols approved by the institutional animal welfare committees and the legal requirements of France. As part of their medical training, individuals have learned to occasionally masturbate and donate their semen for rewards. No human intervention is involved in the collection, the act being carried out spontaneously by the male, without constraint or stress. The male ejaculates in his hand where semen coagulate immediately and exchanges it for a reward (fruits or sweets) from the caretaker who immediately places it in 50 mL tube. Two ejaculates were obtained from first individual and one from the second. In addition, a single blood sample was taken for karyotyping from both individuals as part of a routine annual check-up. One human blood sample was obtained from a donor with a known 46,XY normal karyotype. An informed consent had been signed in accordance with local protocols and the principles of the Declaration of Helsinki. Unless specified otherwise, all reagents were purchased from Sigma-Aldrich.

### 4.2. Karyotype

Blood cultures were carried out in 15 mL Falcon tubes in Chromosome P medium (Euroclone) for 48 h. The cells were blocked in metaphase by addition to the culture of 20 µL of Colcemid (N-Deacetyl-N-methylcolchicine, 10 µg/mL, Gibco, France) for 30 min at 37 °C, then incubate at 37 °C for 20 min in a hypotonic solution to induce plasma membrane destruction. Cells were fixed for 30 min using 3:1 methanol:acetic acid solution and then dropped on slides. G-banding coloration was performed with Giemsa after trypsin exposition and R-banding coloration was performed with Giemsa and BrdU (5-bromodesoxyuridine). Karyotypes were performed on at least 10 mitoses per sample.

### 4.3. Sperm Preparation

The liquid part released from the coagulum is collected after 30 min of liquefaction at 37 °C. Sperm parameters were assessed according to WHO criteria for human semen analysis [59]. Semen pH and sperm concentration were measured. Motility, vitality using eosin-nigrosin staining and morphology using Harris–Shorr staining were evaluated. As the recovery of the ejaculate from the second individual took a long time, only sperm concentration and morphology could be properly evaluated for him.

Semen was then diluted in Sperm Freeze^®^ (FertiPro NV, Beernem, Belgium) at a ratio of 1:0.7, sealed in high security straws (CryoBioSystem, IMV Technologies, Maple Grove, Minnesota, USA) and frozen in nitrogen vapor on a 3 cm polystyrene rack. As freezing does not affect sperm aneuploidy rates [60,61], the thawing control straws were kept together to be processed simultaneously later.

Regarding slides preparation, straws were thawed at 37 °C for 10 min and post-thawing sperm parameters were assessed. Collection samples had to be pooled to obtain a sufficient number of analyzable cells. Cells were then washed twice in PBS 1X, fixed in 3:1 methanol:acetic acid solution, spread onto slides at optimum density to avoid nuclei overlapping and then dried overnight. Afterwards a mild fixation step with 1% PFA in PBS 1X was performed for 10 min at 4 °C to maintain nuclei structure, followed by two washes in 2 SSC solution, a mild decondensation step with 10 mM DTT in 0.1 M Tris-HCl pH 8.0 with 0.1% (v/v) Triton X-100 for 30 min at room temperature to allow FISH probes penetration, two new washes in 2 SSC solution and then an ethanol series for dehydration before hybridization.

### 4.4. DNA Probes

BAC DNAs were extracted and amplified by rolling circle amplification using the bacteriophage phi29 (Kit GE Healthcare, Amersham Biosciences, Buckinghamshire, UK). The quantity and purity of the amplified DNA are evaluated with Nanodrop (Thermo scientific) and a purification step is performed if necessary (DNA Clean and concentrator kit, Ozyme). The purified BACs are then conjugated to fluorochromes (Green-dUTP, Enzo life sciences; Aqua-dUTP, Enzo Life Sciences; Tetramethyl-Rhodamine-5-dUTP, Roche Diagnostics) with a Nick translation Reagent Kit (Abbott Laboratories). The double exonuclease and polymerase activity of the enzyme polymerase I removes the original nucleotides from the DNA matrix and incorporates the labeled deoxynucleotides. The labeled probes are then precipitated by addition of salts (5M NaCl) in an alcoholic medium to reach a high ionic strength and then precipitated at −80 °C for at least 2 h in the presence of human Cot-1 DNA (Invitrogen) to block non-specific hybridizations and suppress repetitive DNA sequences. The supernatant is removed after centrifugation for 20 min at 4 °C and 16,200× *g*, then the pellet is resuspended with hybridization buffer.

### 4.5. FISH and Scoring

First, all selected probes were tested individually on both human and chimpanzee mitosis simultaneously in order to validate their position, size and brightness and verify that no cross-hybridization were present. Analyses were performed on at least 20 mitosis for both species. Then, all spermFISH experiments were done using standard multicolor FISH with sets of validated probes targeting two to three different chromosomes per sample as follows (S, Spectrum; G, Green; O, Orange; A, Aqua): The sets used were 17qSG/16qSO, 12qSG/8qSO, 20qSG/18qSO, 2qSG/7qSO, 4qSG/15qSO, 9qSG/22qSO, 1qSG/19qSO, 11qSG/3qSO, 6qSG/14qSO, 5qSG/2pSO and 21qSA/XqSG/YSO).

Samples were co-denatured with the probe sets for 2 min at 75 °C and then hybridized for 18 h in a HYBrite^®^ system (Abbott Laboratories). Slides are then washed 2 min at 73.2 °C in 0.4 SSC solution with 0.3% NP40 and 1 min at room temperature in 2 SSC with 0.1% NP40, then mounted with DAPI II (Abbott Laboratories) which counterstain sperm nuclei.

Scoring was performed with a METAFER Metasystems^®^ device, previously validated for spermFISH analysis [36]. The galleries of images provided by the machine were manually verified by two experienced technicians (CG and MG) applying the same strict criteria. These strict reading criteria were used for both automated and manual reading: spermatozoa had to be intact with clearly defined border, no swelling and non-overlapping. To be eligible, hybridization signals must be clearly delineated, positioned inside the head, similar in size and brightness, and separated by at least the size of a signal [29,62,63]. If needed, a complementary manual reading was performed to obtain a minimum of 10,000 analyzed cells per sample.

Spermatozoa were considered normal if they presented one distinct signal for each chromosome, disomic if they presented two distinct signals for a chromosome, nullisomic if no signal was detected for only one chromosome in a set, and diploid if they present two distinct signals for all chromosomes investigated. Nuclei without signals were considered as hybridization failure.

## 5. Conclusions

In this study, we provide a first insight into the incidence and distribution of chromosomal aneuploidies in chimpanzee sperm. Our data points out some very exciting questions about aneuploidy and we hope that future additional data from other great apes will lead to further discoveries. To this end, zoos and researchers must continue to collaborate in a common mission to improve the knowledge of wildlife in order to work for its conservation.

## Figures and Tables

**Table 1:** Sperm parameters of both chimpanzees ^†^.

	Individual 1		Individual 2	Literature Range or Mean (*n* = 69) ^†^
Ejaculate	1	2	1	
**Sperm parameters**				
Sperm volume (µL)	600	700	400	100–4400
pH	8	7.9	-	-
Sperm concentration (10^6^/mL)	92	290	65	61–11300
Vitality (% alive)	62	77	-	72.13
**Sperm motility**				
Total motility (%)	55	72	-	3–93.2
Average path velocity (µM/s)	76.70 ± 40.67	107.32 ± 49.12	-	138
Straight linear velocity (µM/s)	58.15 ± 37.26	62.59 ± 48.62	-	34.3
Curvilinear velocity (µM/s)	128.43 ± 50.48	136.70 ± 53.25	-	110.3
Amplitude of lateral head displacement (µM)	7.32 ± 4.19	6.98 ± 4.57	-	5.2
**Sperm morphology**				
Normal morphology (%)	87	83	79	69.58
Head defects (%)	5	8	7	-
Abnormal base (%)	2	3	4	-
Abnormal acrosome (%)	2	4	2	-
Macro head (%)	0	1	0	-
Thinned head (%)	1	0	1	-
Flagellum defects (%)	8	9	14	-
Coiled tail (%)	4	5	6	-
Destructured tail (%)	2	1	1	-
Multiple tail (%)	1	0	0	-
Simple bent tail (%)	1	3	7	-
**Sperm morphometry**				
Head length (µM ± SE)	4.40 ± 0.22	-	-	4.8
Head width (µM ± SE)	2.79 ± 0.19	-	-	2.8
Head area (µM ± SE)	9.87 ± 0.80	-	-	-
Head perimeter (µM ± SE)	11.79 ± 0.44	-	-	-
Head ellipticity (µM ± SE)	1.58 ± 0.12	-	-	1.68
Elongation (µM ± SE)	0.22 ± 0.04	-	-	0.25
Roughness (µM ± SE)	0.89 ± 0.07	-	-	-
Regularity (µM ± SE)	0.98 ± 0.07	-	-	-
Midpiece length (µM ± SE)	5.67 ± 0.56	-	-	6.79
Principal piece length (µM ± SE)	54.03 ± 2.73	-	-	-
Flagellum length (µM ± SE)	59.71 ± 2.75	-	-	52.25
Total length (µM ± SE)	64.11 ± 2.75	-	-	59.30

^†^ Literature data from [9].

**Table 2:** Disomy rates per chromosome in chimpanzee.

CHROMOSOME (PTR)	NUMBER OF DISOMIC CELLS (%)	NUMBER OF NUCLEI ANALYZED
**1**	8 (0.080)	10,027
**2**	5 (0.050)	10,092
**3**	6 (0.060)	10,013
**4**	6 (0.060)	10,003
**5**	8 (0.079)	10,121
**6**	6 (0.059)	10,168
**7**	3 (0.030)	10,059
**8**	2 (0.020)	10,055
**9**	7 (0.069)	10,092
**10**	5 (0.050)	10,059
**11**	8 (0.078)	10,278
**12**	5 (0.050)	10,003
**13**	8 (0.079)	10,168
**14**	5 (0.050)	10,055
**15**	4 (0.040)	10,121
**16**	9 (0.090)	10,013
**17**	2 (0.020)	10,003
**18**	7 (0.070)	10,025
**19**	3 (0.030)	10,025
**20**	7 (0.070)	10,027
**21**	6 (0.060)	10,003
**22**	8 (0.079)	10,108
**23**	4 (0.039)	10,298
**X/Y**	2 (0.198)	10,108

**Table 3:** Diploidy rates per chromosome set.

CHROMOSOME SET (PTR)	NUMBER OF DIPLOID CELLS (%)	NUMBER OF NUCLEI ANALYZED
**18/19**	3 (0.030)	10,025
**7/10**	5 (0.050)	10,059
**17/21**	6 (0.060)	10,003
**6/13**	10 (0.098)	10,168
**3/16**	6 (0.060)	10,013
**11/23**	8 (0.078)	10,298
**1/20**	4 (0.040)	10,027
**2/9**	6 (0.059)	10,092
**5/15**	5 (0.049)	10,121
**4/12**	4 (0.040)	10,003
**8/14**	3 (0.030)	10,055
**22/X/Y**	4 (0.040)	10,108
**MEAN ± SD**	64 (0.053)	120,972

**Table 4:** Interspecies comparison of sperm disomy rates between *Homo sapiens* (HSA, 36 studies), *Pan troglodytes* (PTR, our study, *n* = 2) and *Macaca mulatta* (MMU, one study, *n* = 5).

**HSA**	**1**	**2**		**3**	**4**	**5**	**6**	**7**	**8**	**9**	**10**	**11**	**12**	**13**	**14**	**15**	**16**	**17**	**18**	**19**	**20**	**21**	**22**	**XY**
disomy (%)	0.088	0.089		0.200	0.078	-	0.040	0.050	0.038	0.094	-	-	0.145	0.109	-	0.346	0.209	0.170	0.077	0.430	0.120	0.154	0.370	0.267
**PTR**	**1**	**12**	**13**	**2**	**3**	**4**	**5**	**6**	**7**	**11**	**8**	**9**	**10**	**14**	**15**	**16**	**18**	**19**	**17**	**20**	**21**	**22**	**23**	**XY**
disomy (%)	0.080	0.050	0.079	0.050	0.060	0.060	0.079	0.059	0.030	0.078	0.020	0.069	0.050	0.050	0.040	0.090	0.070	0.030	0.020	0.060	0.060	0.079	0.038	0.197
**MMU**	**1**	**12**	**13**	**2**	**5**	**6**	**4**	**3q**	**8**	**15**	**9**	**14**	**11**	**17**	**7q**	**7p**	**20**	**16**	**18**	**19**	**10q**	**3p**	**10p**	**XY**
disomy (%)	-	-	-	-	-	-	-	-	-	-	-	-	-	0.040	-	-	0.060	0.040	0.040	0.030	0.140	-	-	0.190

## Data Availability

All data that support the findings of this study are available from the corresponding author upon reasonable request.

## Supplementary Materials

The following are available online at https://www.mdpi.com/article/10.3390/ijms221910383/s1, Table S1: List of all BACs used for the constitution of the probe sets for each chromosome. Sizes and positions are given in base pairs for hg18 genome, Table S2: Literature review of sperm aneuploidy in human.

## Abbreviations

ART	assisted reproduction techniques
BAC	bacterial artificial chromosome
df	degrees of freedom
DNA	deoxyribonucleic acid
EAZA	European Association of Zoos and Aquaria
FISH	fluorescence in situ hybridization
HSA	*Homo sapiens*
MMU	*Macaca mulatta*
NOR	nucleolus organizer regions
NS	not significant
PTR	*Pan troglodytes*
SA	spectrum aqua
SE	standard error
SG	spectrum green
SO	spectrum orange
WHO	World Health Organization

## References

[1] Fenster C., Ballou J., Dudash M., Eldridge M., Frankham R., Lacy R., Ralls K., Sunnucks P. (2018). Conservation and Genetics. Yale J. Biol. Med..

[2] International Union for Conservation of Nature—IUCN Website. https://www.iucn.org/fr.

[3] Estrada A., Garber P., Rylands A., Roos C., Fernandez-Duque E., Di Fiore A., Nekaris K., Nijman V., Heymann E., Lambert J. (2017). Impending extinction crisis of the world’s primates: Why primates matter. Sci. Adv..

[4] Osada N. (2015). Genetic diversity in humans and non-human primates and its evolutionary consequences. Genes Genet. Syst..

[5] DeSalle R., Amato G. (2004). The expansion of conservation genetics. Nat. Rev. Genet..

[6] Arandjelovic M., Vigilant L. (2018). Non-invasive genetic censusing and monitoring of primate populations. Am. J. Primatol..

[7] Andrabi S., Maxwell W. (2007). A review on reproductive biotechnologies for conservation of endangered mammalian species. Anim. Reprod. Sci..

[8] Comizzoli P., Holt W. (2019). Breakthroughs and new horizons in reproductive biology of rare and endangered animal species. Biol. Reprod..

[9] Martinez G., Garcia C. (2020). Sexual selection and sperm diversity in primates. Mol. Cell. Endocrinol..

[10] Villagómez D., Pinton A. (2008). Chromosomal abnormalities, meiotic behavior and fertility in domestic animals. Cytogenet. Genome Res..

[11] Villagómez D., Parma P., Radi O., Di Meo G., Pinton A., Iannuzzi L., King W. (2009). Classical and Molecular Cytogenetics of Disorders of Sex Development in Domestic Animals. Cytogenet. Genome Res..

[12] Ramasamy R., Scovell J., Kovac J., Cook P., Lamb D., Lipshultz L. (2015). Fluorescence in situ hybridization detects increased sperm aneuploidy in men with recurrent pregnancy loss. Fertil. Steril..

[13] Raudsepp T., Chowdhary B. (2016). Chromosome Aberrations and Fertility Disorders in Domestic Animals. Annu. Rev. Anim. Biosci..

[14] Rodrigo L. (2020). Sperm genetic abnormalities and their contribution to embryo aneuploidy & miscarriage. Best Pract. Res. Clin. Endocrinol. Metab..

[15] Weiss G., Weick R., Knobil E., Wolman S., Gorstein F. (1973). An X-O Anomaly and Ovarian Dysgenesis in a Rhesus Monkey. Folia Primatol..

[16] Ruppenthal G., Caffery S., Goodlin B., Sackett G., Vigfusson N., Peterson V. (1983). Pigtailed macaques (Macaca nemestrina) with trisomy X manifest physical and mental retardation. Am. J. Ment. Defic..

[17] Reyes F., Osborn R., Fuller G., Hobson W., Greenberg C., Ray M., Thliveris J., Faiman C. (1990). Gonadal dysgenesis with X-monosomy in a cynomolgus monkey (Macaca fascicularis). Am. J. Primatol..

[18] Moore C., Leland M., Brzyski R., McKeand J., Witte S., Rogers J. (1998). A baboon (*Papio hamadryas*) with an isochromosome for the long arm of the X. Cytogenet. Genome Res..

[19] Ruppenthal G., Moore C., Best R., Walker-Gelatt C., Delio P., Sackett G. (2004). Trisomy 16 in a Pigtailed Macaque (M. nemestrina) With Multiple Anomalies and Developmental Delays. Am. J. Ment. Retard..

[20] Dudley C., Hubbard G., Moore C., Dunn B., Raveendran M., Rogers J., Nathanielsz P., McCarrey J., Schlabritz-Loutsevitch N. (2005). A male baboon (*Papio hamadryas*) with a mosaic 43,XXY/42,XY karyotype. Am. J. Med. Genet. Part A.

[21] Hassanane M., Kovacs A., Laurent P., Lindblad K., Gustavsson I. (1999). Simultaneous detection of X- and Y-bearing bull spermatozoa by double colour fluorescence in situ hybridization. Mol. Reprod. Dev..

[22] Nicodemo D., Pauciullo A., Castello A., Roldan E., Gomendio M., Cosenza G., Peretti V., Perucatti A., Di Meo G., Ramunno L. (2009). X-Y Sperm Aneuploidy in 2 Cattle (*Bos taurus*) Breeds as Determined by Dual Color Fluorescent in situ Hybridization (FISH). Cytogenet. Genome Res..

[23] Pauciullo A., Cosenza G., Peretti V., Iannuzzi A., Di Meo G., Ramunno L., Iannuzzi L., Rubes J., Di Berardino D. (2011). Incidence of X-Y aneuploidy in sperm of two indigenous cattle breeds by using dual color fluorescent in situ hybridization (FISH). Theriogenology.

[24] Di Berardino D., Vozdova M., Kubickova S., Cernohorska H., Coppola G., Coppola G., Enne G., Rubes J. (2003). Sexing river buffalo (*Bubalus bubalis* L.), sheep (*Ovis aries* L.), goat (*Capra hircus* L.), and cattle spermatozoa by double color FISH using bovine (*Bos taurus* L.) X- and Y-painting probes. Mol. Reprod. Dev..

[25] Bugno-Poniewierska M., Kozub D., Pawlina K., Tischner M., Tischner M., Słota E., Wnuk M. (2011). Determination of the Correlation Between Stallion’s Age and Number of Sex Chromosome Aberrations in Spermatozoa. Reprod. Domest. Anim..

[26] Bugno M., Jablonska Z., Tischner M., Klukowska-Rötzler J., Pienkowska-Schelling A., Schelling C., Slota E. (2009). Detection of Sex Chromosome Aneuploidy in Equine Spermatozoa Using Fluorescence in Situ Hybridization. Reprod. Domest. Anim..

[27] Rubeš J., Vozdová M., Kubíčková S. (1999). Aneuploidy in pig sperm: Multicolor fluorescence in situ hybridization using probes for chromosomes 1, 10, and Y. Cytogenet. Genome Res..

[28] Orsztynowicz M., Pawlak P., Oleś D., Kubickova S., Lechniak D. (2011). Low incidence of chromosome aberrations in spermatozoa of fertile boars. Reprod. Biol..

[29] Komaki H., Oi M., Suzuki H. (2014). Detection of sex chromosome aneuploidy in dog spermatozoa by triple color fluorescence in situ hybridization. Theriogenology.

[30] O’Brien J., Stojanov T., Heffernan S., Hollinshead F., Vogelnest L., Chis Maxwell W., Evans G. (2005). Flow cytometric sorting of non-human primate sperm nuclei. Theriogenology.

[31] Froenicke L., Hung P., VandeVoort C., Lyons L. (2007). Development of a non-human primate sperm aneuploidy assay tested in the rhesus macaque (*Macaca mulatta*). MHR Basic Sci. Reprod. Med..

[32] Morrell J., Hodges J. (1998). Cryopreservation of non-human primate sperm: Priorities for future research. Anim. Reprod. Sci..

[33] Netten H., Young I., van Vliet L., Tanke H., Vroljik H., Sloos W. (1997). FISH and chips: Automation of fluorescent dot counting in interphase cell nuclei. Cytometry.

[34] Carrell D., Emery B. (2008). Use of automated imaging and analysis technology for the detection of aneuploidy in human sperm. Fertil. Steril..

[35] Molina Ò., Sarrate Z., Vidal F., Blanco J. (2009). FISH on sperm: Spot-counting to stop counting? Not yet. Fertil. Steril..

[36] Martinez G., Gillois P., Le Mitouard M., Borye R., Esquerré-Lamare C., Satre V., Bujan L., Hennebicq S. (2013). FISH and tips: A large scale analysis of automated versus manual scoring for sperm aneuploidy detection. Basic Clin. Androl..

[37] Lammers J., Splingart C., Barrière P., Jean M., Fréour T. (2013). Double-blind prospective study comparing two automated sperm analyzers versus manual semen assessment. J. Assist. Reprod. Genet..

[38] Lee C., Barber G., Casper J., Clawson H., Diekhans M., Gonzalez J., Hinrichs A., Lee B., Nassar L., Powell C. (2019). UCSC Genome Browser enters 20th year. Nucleic Acids Res..

[39] Kasai F., Takahashi E., Koyama K., Terao K., Suto Y., Tokunaga K., Nakamura Y., Hirai M. (2000). Comparative FISH mapping of the ancestral fusion point of human chromosome 2. Chromosome Res. Int. J. Mol. Supramol. Evol. Asp. Chromosome Biol..

[40] Ioannou D., Fortun J., Tempest H. (2018). Meiotic nondisjunction and sperm aneuploidy in humans. Reproduction.

[41] Schmickel R., Gonzalez I., Erickson J. (1985). Nucleolus Organizing Genes on Chromosome 21: Recombination and Nondisjunction. Ann. N. Y. Acad. Sci..

[42] García M., Dietrich A., Pujol R., Egozcue J. (1989). Nucleolar structures in chromosome and SC preparations from human oocytes at first meiotic prophase. Hum. Genet..

[43] Ford J., Lester P. (1982). Factors affecting the displacement of human chromosomes from the metaphase plate. Cytogenet. Genome Res..

[44] Spriggs E., Rademaker A., Martin R. (1996). Aneuploidy in human sperm: The use of multicolor FISH to test various theories of nondisjunction. Am. J. Hum. Genet..

[45] Ferlin A., Garolla A., Foresta C. (2005). Chromosome abnormalities in sperm of individuals with constitutional sex chromosomal abnormalities. Cytogenet. Genome Res..

[46] Garcia-Cruz R., Casanovas A., Brieno-Enriquez M., Robles P., Roig I., Pujol A., Cabero L., Durban M., Garcia Caldes M. (2009). Cytogenetic analyses of human oocytes provide new data on non-disjunction mechanisms and the origin of trisomy 16. Hum. Reprod..

[47] Harton G., Tempest H. (2011). Chromosomal disorders and male infertility. Asian J. Androl..

[48] Uroz L., Templado C. (2012). Meiotic non-disjunction mechanisms in human fertile males. Hum. Reprod..

[49] Tang S., Gao H., Zhao Y., Ma S. (2009). Aneuploidy and DNA fragmentation in morphologically abnormal sperm. Int. J. Androl..

[50] Coban O., Serdarogullari M., Onar Sekerci Z., Bilgin E., Serakinci N. (2018). Evaluation of the impact of sperm morphology on embryo aneuploidy rates in a donor oocyte program. Syst. Biol. Reprod. Med..

[51] Nagaoka S., Hassold T., Hunt P. (2012). Human aneuploidy: Mechanisms and new insights into an age-old problem. Nat. Rev. Genet..

[52] Brieño-Enríquez M., Cohen P. (2015). Double trouble in human aneuploidy. Nat. Genet..

[53] Stevison L., Woerner A., Kidd J., Kelley J., Veeramah K., McManus K., Bustamante C., Hammer M., Wall J. (2015). The Time Scale of Recombination Rate Evolution in Great Apes. Mol. Biol. Evol..

[54] Auton A., Fledel-Alon A., Pfeifer S., Venn O., Segurel L., Street T., Leffler E., Bowden R., Aneas I., Broxholme J. (2012). A Fine-Scale Chimpanzee Genetic Map from Population Sequencing. Science.

[55] Scally A., Dutheil J., Hillier L., Jordan G., Goodhead I., Herrero J., Hobolth A., Lappalainen T., Mailund T., Marques-Bonet T. (2012). Insights into hominid evolution from the gorilla genome sequence. Nature.

[56] Liu C., Liu H., Zhang H., Wang L., Li M., Cai F., Wang X., Wang L., Zhang R., Yang S. (2021). Paternal USP26 mutations raise Klinefelter syndrome risk in the offspring of mice and humans. EMBO J..

[57] García-Ferreyra J., Hilario R., Dueñas J. (2018). High percentages of embryos with 21, 18 or 13 trisomy are related to advanced paternal age in donor egg cycles. JBRA Assist. Reprod..

[58] Robbins W., Elashoff D., Xun L., Jia J., Li N., Wu G., Wei F. (2005). Effect of lifestyle exposures on sperm aneuploidy. Cytogenet. Genome Res..

[59] Martinez G., Walschaerts M., Le Mitouard M., Borye R., Thomas C., Auger J., Berthaut I., Brugnon F., Daudin M., Moinard N. (2017). Impact of Hodgkin or non-Hodgkin lymphoma and their treatments on sperm aneuploidy: A prospective study by the French CECOS network. Fertil. Steril..

[60] Losdat S., Chang S., Reid J. (2014). Inbreeding depression in male gametic performance. J. Evol. Biol..

[61] Zhang M., Zhai L., Fang Z., Li A., Qiu Y., Liu Y. (2018). Impact of a mild scrotal heating on sperm chromosomal abnormality, acrosin activity and seminal alpha-glucosidase in human fertile males. Andrologia.

[62] Perry M., Young H., Grandjean P., Halling J., Petersen M., Martenies S., Karimi P., Weihe P. (2016). Sperm Aneuploidy in Faroese Men with Lifetime Exposure to Dichlorodiphenyldichloroethylene (p,p’-DDE) and Polychlorinated Biphenyl (PCB) Pollutants. Environ. Health Perspect..

[63] Radwan M., Jurewicz J., Sobala W., Brzeźnicki S., Radwan P., Jakubowski L., Hawuła W., Ulańska A., Hanke W. (2016). Human sperm aneuploidy after exposure to polycyclic aromatic hydrocarbons. Reprod. Fertil. Dev..

[64] Governini L., Guerranti C., De Leo V., Boschi L., Luddi A., Gori M., Orvieto R., Piomboni P. (2014). Chromosomal aneuploidies and DNA fragmentation of human spermatozoa from patients exposed to perfluorinated compounds. Andrologia.

[65] Jurewicz J., Radwan M., Wielgomas B., Klimowska A., Kałużny P., Radwan P., Jakubowski L., Hanke W. (2017). Environmental exposure to parabens and sperm chromosome disomy. Int. J. Environ. Health Res..

[66] Perry M. (2008). Effects of environmental and occupational pesticide exposure on human sperm: A systematic review. Hum. Reprod. Update.

[67] Rademaker A., Spriggs E., Ko E., Martin R. (1997). Reliability of estimates of diploid human spermatozoa using multicolour fluorescence in-situ hybridization. Hum. Reprod..

[68] Rubes J., Vozdova M., Oracova E., Perreault S. (2005). Individual variation in the frequency of sperm aneuploidy in humans. Cytogenet. Genome Res..

[69] Tempest H., Ko E., Rademaker A., Chan P., Robaire B., Martin R. (2009). Intra-individual and inter-individual variations in sperm aneuploidy frequencies in normal men. Fertil. Steril..

