# Supplementary material for: FISH and Chimps: Insights into Frequency and Distribution of Sperm Aneuploidy in Chimpanzees (Pan troglodytes)
Source: Int J Mol Sci. 2021 Sep 27;22(19):10383. doi: 10.3390/ijms221910383 (PMC8509033; doi:10.3390/ijms221910383)
Supplement: Supplementary file 1 [file ijms-22-10383-s001.zip › ijms-1386271-SI.pdf]

SUPPLEMENTAL DATA

Supplemental Table S1. List of all BACs used for the constitution of the probe sets for each chromosome. Sizes and positions are given in base pairs for hg18 genome.

Probe	Target chromosomes	Total size (bp)	BAC identification	Position (bp)	Size (bp)
**1q**	*HSA* 1 / *PTR* 1	1,127,322	RP11-438F14	246754133-246932000	177,867
			CTD-3000C19	246529431-246722177	192,746
			RP11-469H22	246250426-246455373	204,947
			RP11-908P10	245985930-246193139	207,209
			CTD-2548B21	244064096-244262971	198,875
**2p**	*HSA* 2 / *PTR* 12	520,649	GS1-68F18	224263-403255	178,992
			RP11-125K7	596981-744912	147,931
**2q**	*HSA* 2 / *PTR* 13	1,076,262	RP11-351E10	242375991-242565755	189,764
			RP11-875C22	242147376-242359508	212,132
			RP11-90E11	242009527-242187120	177,593
			RP11-952H3	241827539-242014017	186,478
			RP11-185C8	241680132-241864592	184,460
			RP11-143H14	241489493-241648076	158,583
**3q**	*HSA* 3 / *PTR* 2	1,918,615	RP11-159K3	199038951-199230435	191,484
			CTD-2529O9	198655574-198826526	170,952
			RP11-114F20	198339452-198528164	188,712
			RP11-183C22	198146622-198339454	192,832
			RP11-927L5	197896915-198070611	173,696
			RP11-200I19	197700433-197837659	137,226
			RP11-106N22	197589202-197656469	67,267
			RP11-447L10	197402231-197591199	188,968
			RP11-185G19	197311820-197404234	92,414
**4q**	*HSA* 4 / *PTR* 3	1,114,662	RP11-45F23	190520006-190668423	148,417
			RP11-256N12	190366130-190552194	186,064
			RP11-463B4	190179198-190387639	208,441
			RP11-354H17	189958830-190151921	193,091
			RP11-1077O22	189778946-189960823	181,877
			RP11-636B14	189553761-189739148	185,387
**5q**	*HSA* 5 / *PTR* 4	1,149,052	RP11-69N15	180429200-180616147	186,947
			CTD-2530D22	180124020-180396206	272,193
			RP11-451H23	179870132-180060155	190,023
			RP11-282I19	179629463-179805650	176,187
			RP11-252I14	179467095-179662550	195,455
**6q**	*HSA* 6 / *PTR* 5	1,010,693	RP11-614P3	169746523-169946788	200,265
			RP11-369H17	169584276-169746513	162,237
			RP11-135E20	169430897-169619248	188,351
			RP11-417E7	169221863-169333040	111,177
			RP11-35J6	168936095-169093666	157,571
**7q**	*HSA* 7 / *PTR* 6	1,238,519	RP11-1112M14	158602180-158788150	185,970
			RP11-664B5	158404470-158595970	191,500
			CTD-2554I6	158081863-158344153	262,298
			CTD-2033H20	157802288-158058234	255,946
			RP11-11B21	157752948-157964627	211,679
			RP11-518I12	157549631-157754947	205,316

**8q**	*HSA* 8 / *PTR* 7	1,128,540	RP11-590B21	145963321-146131249	167,928
			RP11-1143I12	145825257-145978282	153,025
			RP11-620H1	145536611-145740218	203,607
			CTD-3232M19	145403397-145494495	91,098
			CTD-3065J16	145002709-145218050	215,341
**9q**	*HSA* 9 / *PTR* 9	1,256,908	RP11-937L7	139785404-139993460	208,056
			RP11-417A4	139523178-139716008	192,830
			RP11-48C7	139459573-139634567	174,994
			CTD-2377P2	139297032-139429236	132,220
			RP11-350O14	139029415-139220879	191,464
			RP11-673E5	138946390-139158292	211,902
			RP11-769N4	138736552-138946296	209,744
			CTD-2551F21	138554306-138754280	199,974
**10q**	*HSA* 10 / *PTR* 8	1,149,860	RP11-108K14	135078861-135240498	161,637
			RP11-122K13	134955982-135072292	116,310
			RP11-1022E21	134703784-134906603	202,819
			RP11-97M24	134607245-134778110	170,865
			RP11-288G11	134340940-134549003	208,063
			RP11-500B2	134228392-134341039	112,647
			RP11-384O10	134090638-134276997	186,359
**11q**	*HSA* 11 / *PTR* 9	1,129,412	RP11-410I24	133413709-134301418	887,709
			RP11-469N6	133983990-134156497	172,507
			RP11-627G23	133832598-133898497	65,899
			RP11-164K8	133527689-133732868	205,179
			RP11-368H5	133348982-133518619	169,637
			RP11-259H11	133172006-133349688	177,682
**12q**	*HSA* 12 / *PTR* 10	1,413,614	CTD-2140B24	132128913-132289534	160,621
			RP11-386I8	132034460-132178738	144,278
			RP11-46H11	131855460-132036476	181,016
			CTD-3237H24	131516349-131759664	243,315
			RP11-867C16	131361271-131545666	184,395
			CTD-2519K8	131054254-131272060	217,806
			CTD-2522P9	130875920-131078105	202,192
**13q**	*HSA* 13 / *PTR* 14	1,325,533	RP11-569D9	113930807-114103243	172,436
			RP11-245B11	113770458-113932864	162,406
			RP11-199F6	113473995-113644014	170,019
			RP11-230F18	113166605-113351493	184,888
			RP11-391H12	112999602-113025743	26,141
			RP11-98F14	112777710-112939870	162,160
**14q**	*HSA* 14 / *PTR* 15	1,306,717	RP11-417P24	105267349-105437150	169,801
			RP11-731F5	105018846-105195083	176,237
			RP11-435F10	104781176-104980676	199,500
			RP11-1087P8	104677732-104876215	198,483
			RP11-44N21	104552642-104711108	158,466
			RP11-18C13	104381504-104533148	151,644
			RP11-982M15	104130433-104336369	205,936
**15q**	*HSA* 15 / *PTR* 16	1,017,092	RP11-829L22	99860523-100046963	186,440
			RP11-530H6	99746892-99914703	167,811
			RP11-299F21	99550365-99734009	183,644
			CTD-2502E10	99316896-99544556	227,660

			CTD-3211D11	99029871-99262670	232,799
**16q**	*HSA* 16 / *PTR* 18	1,082,241	RP11-417N6	88466848-88643471	176,623
			RP11-7D23	88297302-88297857	169 540
			RP11-1089G18	88081018-88285940	204,922
			RP11-880I20	87847056-88067286	220,230
			RP11-1122C1	87721624-87875044	153,420
			RP11-933C23	87561230-87724152	162,922
**17q**	*HSA* 17 / *PTR* 19	1,494,456	CTD-2519K10	78335862-78488483	457,855
			RP11-388C12	78200000-78311473	111,473
			RP11-525L23	77424566-78592153	1,167,587
			RP11-1087N2	77771385-78520665	749,280
			RP11-51H16	77524868-77690741	165,873
			RP11-634L10	77390102-77562577	172,475
			RP11-765O14	76994027-77189725	195,698
**18q**	*HSA* 18 / *PTR* 17	1,182,640	RP11-565D23	75936873-76103217	166,344
			RP11-93F7	75790939-75945751	154,812
			CTD-3110E17	75603460-75757760	154,300
			RP11-803N2	75432037-75603441	171,404
			RP11-196B3	75261845-75424109	162,264
			RP11-841P22	75089275-75328424	239,149
			RP11-767J19	74920577-75083631	163,054
**19q**	*HSA* 19 / *PTR* 20	1,030,032	CTD-2575K21	63638468-63770533	132,065
			RP11-357E24	63445258-63638426	193,168
			RP11-706G10	63385977-63711682	325,705
			RP11-91H11	63223240-63387841	164,601
			CTD-2583A14	63048608-63182459	133,851
			RP11-1069H17	62854921-63042914	187,993
			RP11-103G13	62608394-62794238	185,844
**20q**	*HSA* 20 / *PTR* 21	1,474,611	CTD-2022N21	62215516-62317284	101,768
			CTD-2559F2	61924110-62120608	196 498
			CTD-3231H17	61807905-61974060	166,155
			RP11-95N13	61564623-61727698	163,075
			RP11-261N11	61332296-61516517	184,221
			CTD-3051D12	61038964-61249909	210,945
			RP11-477N6	60842673-61032192	189,519
**21q**	*HSA* 21 / *PTR* 22	1,101,746	RP11-1000I21	46743970-46940213	196,243
			CTD-3217A14	46620980-46833176	212,196
			RP11-34P17	46391180-46582695	191,515
			RP11-640F21	46144350-46311763	167,413
			RP11-892E8	46068771-46280397	211,626
			RP11-93F5	45979578-46151974	172,396
			RP11-48G23	45838467-46022220	183,753
**22q**	*HSA* 22 / *PTR* 23	827,167	RP11-825H3	49161956-49345964	184,008
			CTD-2579L10	48886805-49061533	174,728
			RP11-931F19	48767143-48969501	202,358
			RP11-49N13	48518797-48673310	154,519
**Xq**	*HSA* X / *PTR* X	1,309,074	RP11-1087L19	153404680-153547872	143,192
			RP11-296N8	153783896-153962641	179,858
			RP11-402H20	153861566-154041424	179,858
			RP11-218L14	154334471-154482330	147,859

			GS1-225F6	154613754-154713754	100,000
**Yq**	*HSA* Y / *PTR* Y	1,542,400	RP11-333E9	12581761-12759800	178,039
			CTD-2191D23	13455167-13658084	202,917
			RP11-386L3	13952205-14124161	171,956

International Journal of *Molecular Sciences*

MDPI

Supplemental Table S2. Literature review of sperm disomy rates in normal man. Results are presented in percentage of disomy per chromosome. Only multicolor-FISH studies with a minimum of 5 donors, strict scoring criteria, and a minimum of 10,000 nuclei counted per probe set were included.

**Reference**	**No. of individuals**	**% disomy**					
		**1**	**2**	**3**	**4**	**6**	**7**
Martin et al., 1995 [75]	10	0.11					
Griffin et al., 1995 [76]	24						
Spriggs et al., 1995, 1996 [44-77]	5	0.09	0.08		0.11		
Downie et al., 1997 [78]	10			0.2			0.05
Lähdetie et al., 1997 [79]	18	0.1					0.06
Kinakin et al., 1997 [80]	18						
Blanco et al., 1997 [81]	5						
Scarpato et al., 1998 [82]	5		0.09				
McInnes et al., 1998 [83]	18	0.09					
Martínez-Pasarell et al., 1999 [84]	8					0.04	
Martin and Rademaker, 1999 [85]	5						
Nishikawa et al., 2000 [86]	5						
Estop et al., 2000 [87]	7						
Shi and Martin, 2000 [88]	10						
Rives et al., 2000 [89]	5						
Bosch et al., 2001, 2003 [90-91]	18					0.04	
Härkönen et al., 2001 [92]	5	0.07					0.06
Soares et al., 2001 [93]	9				0.06		
De Mas et al., 2001 [94]	5						0.04
Hristova et al., 2002 [95]	10						
Ong et al., 2002 [96]	20						
Naccarati et al., 2003 [97]	11		0.09				
Rodrigo et al., 2004 [98]	5						
Xia et al., 2004 [99]	18						
Rubes et al., 2005 [68]	10						
Wyrobek et al., 2006 [100]	90						
Kirkpatrick et al., 2008 [101]	9						
Sun et al., 2008 [102]	6	0.07					
Tempest et al., 2009 [69]	10						
Tang et al., 2010 [54]	9						
Sarrate et al., 2010 [103]	6						
Gordeeva et al., 2011 [104]	11						
Vozdova et al., 2012, 2013 [105-106]	10						0.0315
Vendrell et al., 2013 [107]	6						
Ramasamy et al., 2015 [12]	5						
Garcia-Mengual et al., 2019 [108]	14	0.07	0.09				
**Weighted means values**		**0.088**	**0.088**	**0.2**	**0.078**	**0.04**	**0.05**

References

12. Ramasamy, R.; Scovell, J.; Kovac, J.; Cook, P.; Lamb, D.; Lipshultz, L. Fluorescence in situ hybridization detects increased sperm aneuploidy in men with recurrent pregnancy loss. *Fertil. Steril.* **2015**, *103*, 906–909.e1, doi:10.1016/j.fertnstert.2015.01.029.
44. Spriggs, E.; Rademaker, A.; Martin, R. Aneuploidy in human sperm: The use of multicolor FISH to test various theories of non-disjunction. *Am. J. Hum. Genet.* **1996**, *58*, 356–362.
54. Auton, A.; Fledel-Alon, A.; Pfeifer, S.; Venn, O.; Segurel, L.; Street, T.; Leffler, E.; Bowden, R.; Aneas, I.; Broxholme, J.; et al. A Fine-Scale Chimpanzee Genetic Map from Population Sequencing. *Science* **2012**, *336*, 193–198, doi:10.1126/science.1216872.
68. Rubes, J.; Vozdova, M.; Oracova, E.; Perreault, S. Individual variation in the frequency of sperm aneuploidy in humans. *Cytogenet. Genome Res.* **2005**, *111*, 229–236, doi:10.1159/000086893.
69. Tempest, H.; Ko, E.; Rademaker, A.; Chan, P.; Robaire, B.; Martin, R. Intra-individual and inter-individual variations in sperm aneuploidy frequencies in normal men. *Fertil. Steril.* **2009**, *91*, 185–192, doi:10.1016/j.fertnstert.2007.11.002.
75. Martin, R.; Spriggs, E.; Ko, E.; Rademaker, A. The relationship between paternal age, sex ratios, and aneuploidy frequencies in human sperm, as assessed by multicolor FISH. *Am. J. Hum. Genet.* **1995**, *57*, 1395–1399.
76. Griffin, D.; Abruzzo, M.; Millie, E.; Sheean, L.; Feingold, E.; Sherman, S.; et al. Non-disjunction in human sperm: Evidence for an effect of increasing paternal age. *Hum. Mol. Genet.* **1995**, *4*, 2227–2232, doi:10.1093/hmg/4.12.2227.
77. Spriggs, E.; Rademaker, A.; Martin, R. Aneuploidy in human sperm: Results of two- and three-color fluorescence in situ hybridization using centromeric probes for chromosomes 1 12, 15, 18, X, and Y. *Cytogenet. Genome Res.* **1995**, *71*, 47–53, doi:10.1159/000134060.
78. Downie, S.; Flaherty, S.; Swann, N.; Matthews, C. Estimation of aneuploidy for chromosomes 3, 7, 16, X and Y in spermatozoa from 10 normospermic men using fluorescence in-situ hybridization. *Mol. Hum. Reprod.* **1997**, *3*, 815–819, doi:10.1093/molehr/3.9.815.
79. Lähdetie, J.; Saari, N.; Ajosenpää-Saari, M.; Mykkänen, J. Incidence of aneuploid spermatozoa among infertile men studied by multicolor fluorescence in situ hybridization. *Am. J. Med Genet.* **1997**, *71*, 115–121.
80. Kinakin, B.; Rademaker, A.; Martin, R. Paternal age effect of YY aneuploidy in human sperm, as assessed by fluorescence in situ hybridization. *Cytogenet. Genome Res.* **1997**, *78*, 116–119, doi:10.1159/000134641.
81. Blanco, J.; Rubio, C.; Simon, C.; Egozcue, J.; Vidal, F. Increased incidence of disomic sperm nuclei in a 47,XYY male assessed by fluorescent in situ hybridization (FISH). *Hum. Genet.* **1997**, *99*, 413, doi:10.1007/s004390050381.
82. Scarpato, R.; Naccarati, A.; Mariani, M.; Migliore, L. Aneuploidy and diploidy rates in sperm of five men after three-colour hybridization: Indication of X chromosome-associated autosome 2 aneuploidy. *Mutat. Res. Genet. Toxicol. Environ. Mutagenes.* **1998**, *412*, 227–233, doi:10.1016/s1383-5718(97)00188-5.
83. McInnes, B.; Rademaker, A.; Martin, R. Donor age and the frequency of disomy for chromosomes 1, 13, 21 and structural abnormalities in human spermatozoa using multicolour fluorescence in-situ hybridization. *Hum. Reprod.* **1998**, *13*, 2489–2494, doi:10.1093/humrep/13.9.2489.
84. Martínez-Pasarell, O.; Nogués, C.; Bosch, M.; Egozcue, J.; Templado, C. Analysis of sex chromosome aneuploidy in sperm from fathers of Turner syndrome patients. *Hum. Genet.* **1999**, *104*, 345–349, doi:10.1007/s004390050964.
85. Martin, R.; Rademaker, A. Nondisjunction in human sperm: Comparison of frequencies in acrocentric chromosomes. *Cytogenet. Genome Res.* **1999**, *86*, 43–45, doi:10.1159/000015427.
86. Nishikawa, N.; Murakami, I.; Ikuta, K.; Suzumori, K. *J. Assist. Reprod. Genet.* **2000**, *17*, 97–102, doi:10.1023/a:1009413916753.
87. Estop, A.; Cieply, K.; Munne, S.; Surti, U.; Wakim, A.; Feingold, E. Is there an interchromosomal effect in reciprocal translocation carriers? Sperm FISH studies. *Hum. Genet.* **2000**, *106*, 517–524, doi:10.1007/s004390000275.
88. Shi, Q.; Martin, R. Spontaneous frequencies of aneuploid and diploid sperm in 10 normal Chinese men: Assessed by multicolor fluorescence in situ hybridization. *Cytogenet. Genome Res.* **2000**, *90*, 79–83, doi:10.1159/000015668.
89. Rives, N.; Joly, G.; Machy, A.; Siméon, N.; Leclerc, P.; Macé, B. Assessment of sex chromosome aneuploidy in sperm nuclei from 47,XXY and 46,XY/47,XXY males: Comparison with fertile and infertile males with normal karyotype. *Mol. Hum. Reprod.* **2000**, *6*, 107–112, doi:10.1093/molehr/6.2.107.
90. Bosch, M.; Rajmil, O.; Martínez-Pasarell, O.; Egozcue, J.; Templado, C. Linear increase of diploidy in human sperm with age: A four-colour FISH study. *Eur. J. Hum. Genet.* **2001**, *9*, 533–538, doi:10.1038/sj.ejhg.5200659.
91. Bosch, M.; Rajmil, O.; Egozcue, J.; Templado, C. Linear increase of structural and numerical chromosome 9 abnormalities in human sperm regarding age. *Eur. J. Hum. Genet.* **2003**, *11*, 754–759, doi:10.1038/sj.ejhg.5201049.
92. Harkonen, K.; Suominen, J.; Lahdetie, J. Aneuploidy in spermatozoa of infertile men with teratozoospermia. *Int. J. Androl.* **2001**, *24*, 197–205, doi:10.1046/j.1365-2605.2001.00280.x.
93. Soares, S.; Vidal, F.; Bosch, M.; Martínez-Pasarell, O.; Nogués, C.; Egozcue, J.; Templado, C. Acrocentric chromosome disomy is increased in spermatozoa from fathers of Turner syndrome patients. *Hum. Genet.* **2001**, *108*, 499–503, doi:10.1007/s004390100521.
94. De Mas, P.; Daudin, M.; Vincent, M. Increased aneuploidy in spermatozoa from testicular tumour patients after chemotherapy with cisplatin, etoposide and bleomycin. *Hum. Reprod.* **2001**, *16*, 1204–1208, doi:10.1093/humrep/16.6.1204.
95. Hristova, R.; Ko, E.; Greene, C.; Rademaker, A.; Chernos, J.; Martin, R. Chromosome Abnormalities in Sperm from Infertile Men with Asthenoteratozoospermia1. *Biol. Reprod.* **2002**, *66*, 1781–1783, doi:10.1095/biolreprod66.6.1781.
96. Ong, T.; Xun, L.; Perreault, S.; Robbins, W. Aneuploidy and chromosome breakage in swim-up versus unprocessed semen from twenty healthy men. *J. Androl.* **2002**, *23*, 270–277.

97. Naccarati, A.; Zanello, A.; Landi, S.; Consigli, R.; Migliore, L. Sperm-FISH analysis and human monitoring: A study on workers occupationally exposed to styrene. *Mutat. Res. Genet. Toxicol. Environ. Mutagenes.* **2003**, *537*, 131–140, doi:10.1016/s1383-5718(03)00081-0.
98. Rodrigo, L.; Rubio, C.; Mateu, E.; Simón, C.; Remohí, J.; Pellicer, A.; Gil-Salom, M. Analysis of chromosomal abnormalities in testicular and epididymal spermatozoa from azoospermic ICSI patients by fluorescence in-situ hybridization. *Hum. Reprod.* **2004**, *19*, 118–123, doi:10.1093/humrep/deh012.
99. Xia, Y.; Bian, Q.; Xu, L.; Cheng, S.; Song, L.; Liu, J.; Wu, W.; Wang, S.; Wang, X. Genotoxic effects on human spermatozoa among pesticide factory workers exposed to fenvalerate. *Toxicology* **2004**, *203*, 49–60, doi:10.1016/j.tox.2004.05.018.
100. Wyrobek, A.; Eskenazi, B.; Young, S.; Arnheim, N.; Tiemann-Boege, I.; Jabs, E.; Glaser, R.; Pearson, F.; Evenson, D. Advancing age has differential effects on DNA damage, chromatin integrity, gene mutations, and aneuploidies in sperm. *Proc. Natl. Acad. Sci. USA* **2006**, *103*, 9601–9606, doi:10.1073/pnas.0506468103.
101. Kirkpatrick, G.; Ferguson, K.; Gao, H.; Tang, S.; Chow, V.; Yuen, B.; Ma, S. A comparison of sperm aneuploidy rates between infertile men with normal and abnormal karyotypes. *Hum. Reprod.* **2008**, *23*, 1679–1683, doi:10.1093/humrep/den126.
102. Sun, F.; Mikhaail-Philips, M.; Oliver-Bonet, M.; Ko, E.; Rademaker, A.; Turek, P.; Martin, R. The relationship between meiotic recombination in human spermatocytes and aneuploidy in sperm. *Hum. Reprod.* **2008**, *23*, 1691–1697, doi:10.1093/humrep/den027.
103. Sarrate, Z.; Vidal, F.; Blanco, J. Role of sperm fluorescent in situ hybridization studies in infertile patients: Indications, study approach, and clinical relevance. *Fertil. Steril.* **2010**, *93*, 1892–1902, doi:10.1016/j.fertnstert.2008.12.139.
104. Gordeeva, E.; Shileĭko, L.; Pankratova, O.; Kurilo, L. Aneuploidy frequency in sperm of fertile men. *Genetika* **2011**, *47*, 828–835.
105. Vozdova, M.; Kasikova, K.; Oracova, E.; Prinosilova, P.; Rybar, R.; Horinova, V.; Gaillyova, R.; Rubes, J. The effect of the swim-up and hyaluronan-binding methods on the frequency of abnormal spermatozoa detected by FISH and SCSA in carriers of balanced chromosomal translocations. *Hum. Reprod.* **2012**, *27*, 930–937, doi:10.1093/humrep/der445.
106. Vozdova, M.; Oracova, E.; Kasikova, K.; Prinosilova, P.; Rybar, R.; Horinova, V.; Gaillyova, R.; Rubes, J. Balanced chromosomal translocations in men: Relationships among semen parameters, chromatin integrity, sperm meiotic segregation and aneuploidy. *J. Assist. Reprod. Genet.* **2013**, *30*, 391–405, doi:10.1007/s10815-012-9921-9.
107. Vendrell, X.; Ferrer, M.; García-Mengual, E.; Muñoz, P.; Triviño, J.; Calatayud, C.; Rawe, V.; Ruiz-Jorro, M. Correlation between aneuploidy, apoptotic markers and DNA fragmentation in spermatozoa from normozoospermic patients. *Reprod. Biomed. Online* **2014**, *28*, 492–502, doi:10.1016/j.rbmo.2013.12.001.
108. García-Mengual, E.; Triviño, J.; Sáez-Cuevas, A.; Bataller, J.; Ruíz-Jorro, M.; Vendrell, X. Male infertility: Establishing sperm aneuploidy thresholds in the laboratory. *J. Assist. Reprod. Genet.* **2019**, *36*, 371–381, doi:10.1007/s10815-018-1385-0.
